# The change of serum tumor necrosis factor alpha in patients with type 1 diabetes mellitus: A systematic review and meta-analysis

**DOI:** 10.1371/journal.pone.0176157

**Published:** 2017-04-20

**Authors:** Yong-chao Qiao, Yin-ling Chen, Yan-hong Pan, Fang Tian, Yan Xu, Xiao-xi Zhang, Hai-lu Zhao

**Affiliations:** 1 Center of Diabetic Systems Medicine, Guangxi Key Laboratory of Excellence, Guilin Medical University, Guilin, China; 2 Department of Immunology, Xiangya School of Medicine, Central South University, Changsha, Hunan, China; 3 Department of Immunology, Faculty of Basic Medicine, Guilin Medical University, Guilin, China; Hokkaido Daigaku, JAPAN

## Abstract

**Objective:**

The aim of this study was used meta-analysis to investigate changes of serum tumor necrosis factor-alpha (TNF-α) in patients with type 1 diabetes mellitus (T1DM).

**Methods:**

Relevant literatures were identified from PubMed, Cochrane Library, CNKI, WanFang and Chinese-Cqvip databases (published from January 1, 1999 to September 30, 2016). Eligible reports were included for pooled analysis of serum TNF-α level and subgroup analysis was performed in relation with age, disease duration and ethnicity.

**Results:**

A total of 23 articles (1631 T1DM cases, 1429 healthy controls) were included for this meta-analysis. Compared with the controls, the patients had significantly increased serum TNF-α level (*P* < 0.001). Similar results were also found among all subgroup analysis of different age, disease duration and ethnicity (with the exception of Asian) (all *P* < 0.05). Regression analysis indicated that age (*P* = 0.680), disease duration (*P* = 0.957), and ethnicity (*P* = 0.526) of patients were not significant impact factors for the high heterogeneity. The results were stable according to the sensitivity analysis and no publication bias existed in this meta-analysis.

**Conclusions:**

Serum TNF-α level in T1DM patients has significantly elevated among all age, disease duration and ethnicity groups.

## Introduction

Type 1 diabetes mellitus (T1DM) is a systemic disease leading to abnormal fat, carbohydrate, and protein metabolism due to insulin deficiency [1]. Metabolic proinflammatory disorder, such as chronic hyperglycemia and increased levels of circulating cytokines, suggests immunological disturbances [2–5], which seriously affects the quality of life of the patients and imposes a large economic burden on the national health care system [6]. The reasons responsible for this disease are almost summarized as genetic and environmental factors.

The role of inflammation in diabetes mellitus (DM) has recently been implicated [7] and that inflammatory reaction, mediated by acute phase proteins and cytokines, could lead to the prevention or promotion of diabetes [8, 9]. Inflammatory cytokines such as interleukine-6 (IL-6) [10, 11], IL-17 [12, 13], transforming growth factor-beta (TGF-β) [14] and C-reactive protein (CRP) [15] have been shown to be elevated in DM patients, and the elevated cytokines play an important role in the development and progression of cardiovascular complications.

Tumor necrosis factor-alpha (TNF-α) produced by activated macrophages, CD4+ lymphocytes, natural killer cells, neutrophils, mast cells, eosinophils and neurons, is a cytokine involved in systemic inflammation and always results in acute phase reaction [16]. TNF-α may induce insulin resistance through direct effects on the insulin signaling pathway, and thus participates in the pathogenesis of type 2 DM and obesity [17–20]. As an endogenous factor, TNF-α not only influences energy balance, but also is associated with weight loss, hypermetabolism and resting energy expenditure in malignant diseases [21]. Many researchers focus on the change of serum TNF-α level [21–26] in DM patients, yet findings are inconsistent. In the present study, we performed a pooled analysis of data to define the change of serum TNF-α in T1DM patients.

## Methods

### Search strategies

This study was performed based on the Preferred Reporting Items for Systematic Reviews and Meta-analysis (PRISMA) criteria [27]. The work described here was performed in accordance with the Declaration of Helsinki. This study was approved by the Ethical Committee of Guilin Medical University. We systematically searched five databases (PubMed, Cochrane Library, CNKI, WanFang and Chinese-Cqvip) about the studies published from January 1, 1999 to September 30, 2016. The search strategy using medical terms as following: (“tumor necrosis factor alpha” or “TNF-α”) and (“type 1 diabetes” or “diabetic patients” or “diabetes mellitus” or “DM” or “T1DM”). Otherwise, we identified the additional reports through references cited in recruited articles.

### Inclusion criteria and exclusion criteria

All related articles were reviewed using the criteria as follows: (1) Studies focusing on the change of serum TNF-α level in T1DM patients; (2) Case-control research; (3) Patients used insulin alone; (4) Data expressed as Mean ± SD; and (5) Definitions of T1DM met the criteria recommended by the World Health Organization [28].

Exclusion criteria: (1) For duplicated studies and reports, we only included the latest paper into our final analysis; (2) Animal studies, reviews, editorials, case reports, and personal experience summaries; (3) No healthy controls in the study; (4) Original data displayed as figures or no original data reported; and (5) Inconsistent with the inclusion criteria as described above.

### Quality assessment and data extraction

The Newcastle-Ottawa Scale (NOS) was used to assess the quality of all eligible studies [29] and the following information from each eligible study was extracted independently by two reviewers: (1) first author’s surname; (2) date of publication; (3) country of the studied population; (4) mean age of patients; (5) sample size of case-control; (6) mean disease duration of patients; (7) Mean ± SD displayed the level of serum TNF-α. Considering disagreement, we invited the third investigator to assess such articles through discussion.

### Statistical analysis

We extracted the data (sample size, Mean ± SD) to clarify the change of TNF-α in T1DM patients versus controls, followed by Chi-squared Q test and I2 statistics to estimate the heterogeneity [30, 31]. When *P* < 0.1 or I2 > 50%, we selected a random-effect model to account for possible heterogeneity between studies; otherwise a fixed-effect model was used in the absence of heterogeneity [32, 33]. In order to evaluate the age and disease duration impact on serum TNF-α, all the patients in the included studies were stratified into three groups by age (<12, 12–24, >24 years old) and by disease duration (<5, 5–10, >10 years) respectively. And the impact of different ethnicity of patients was explored by subgroup analysis of five ethnicity groups (Asia, Europe, America, Africa and Oceania). Regression analysis was used to investigate sources of heterogeneity. In addition, we conducted sensitivity analysis by excluding individual studies or selecting articles with high NOS score (≥) or changing the Cochran’s Q statistic methods to check the stability of the results. Publication bias was judged by an Egger’s test (*P* < 0.05 was considered representative of statistically significant publication bias) [34]. Stata 12.0 software was used in this pooled analysis.

## Results

### The process and results of selection

**Fig 1:**
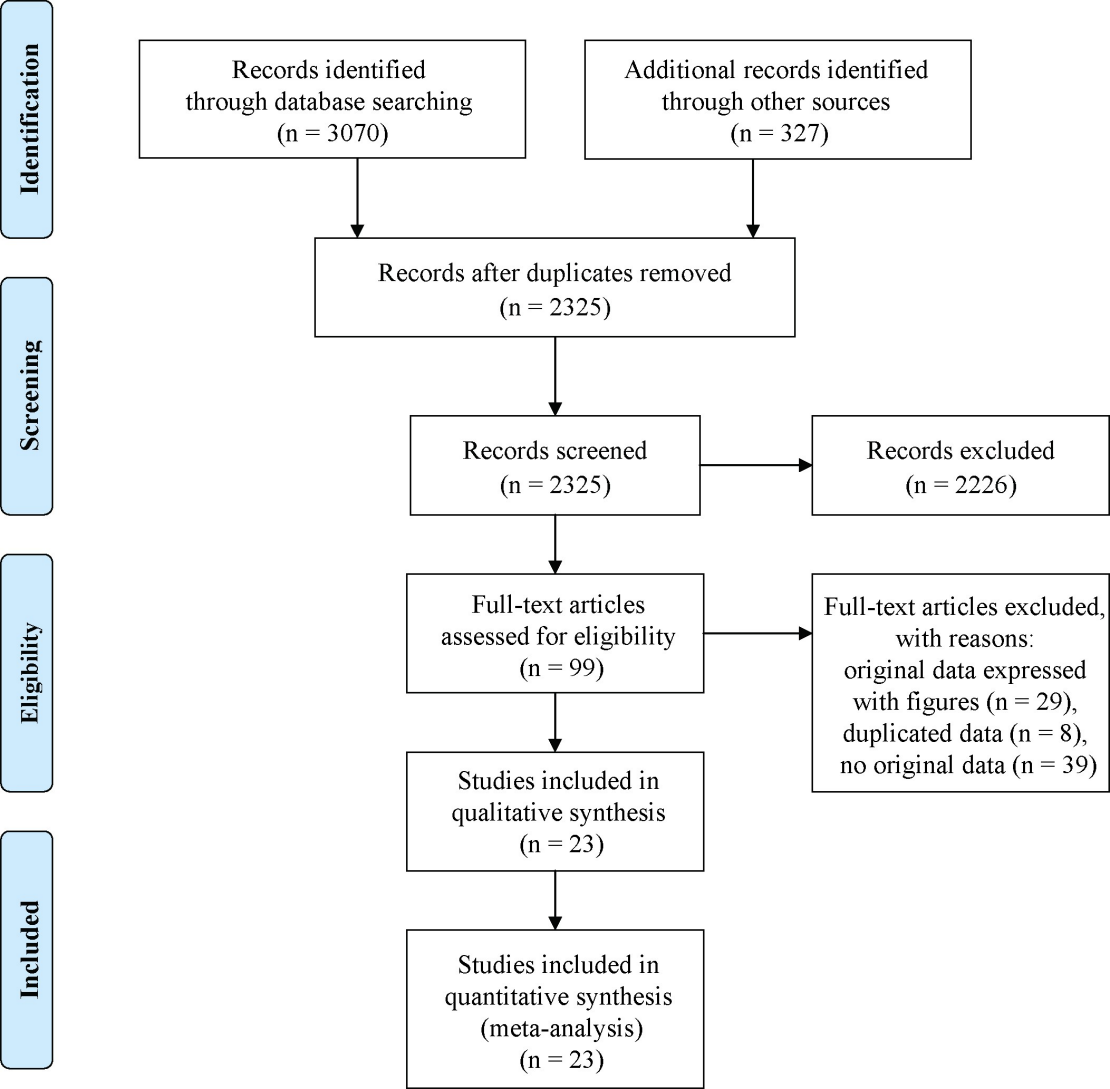
The flow chart of the articles search and inclusion process.

The study selection process was displayed in Fig 1. With our search criteria, we collected 3397 potential studies and excluded 1074 due to duplication. After reading the titles and abstracts, 2226 articles were excluded for no controls, not DM relevant, review and editorial articles or animal studies. Then, we excluded 76 articles due to original data expressed with figures, duplicated data, or no original data. Finally, 20 articles (23 case-control studies) met the criteria and were included in this meta-analysis [1, 7, 21–25, 35–47] (S1 Appendix), involving 1631 T1DM patients and 1429 healthy controls. The specific characteristics of the 23 studies are shown in Table 1.

**Table 1:** Characteristics of studies about serum TNF-α (ng/L) included in this meta-analysis.

Author	Year	Country	Quantification Methods	Duration (years)	Case					Control					NOS score
					SZ	M/F	Age	Mean	SD	SZ	M/F	Age	Mean	SD	
Hegazy[35]	2013	Egypt	ELISA	4.3±2.1	15	7/8	11.1±2.3	9	0.9	15	7/8	11.5±1.4	5.4	1.7	8
Hegazy[35]	2013	Egypt	ELISA	4.4±3.0	15	7/8	11.9±1.4	9.1	0.9	15	7/8	11.5±1.4	5.4	1.7	8
Yuan[36]	2010	China	ELISA	NR	15	NR	18.53±8.70	36.47	16.33	90	NR	28.33±6.12	35.37	14.97	6
Poplawska[1]	2014	Poland	ELISA	NR	62	NR	42.6±12.7	1.6	1.2	6	NR	48.3±2.1	0.8	0.5	6
Talaat[37]	2016	Saudi Arabia	FCM	3.50±0.39	250	NR	8.50±0.5	19.81	8.75	250	NR	8.50±0.5	3.22	0.45	7
Balic ^a^ [38]	2009	Chile	ELISA	0.21±0.12	300	158/142	10.8±4.1	2.31	36.8	310	146/164	11.0±2.2	1.39	0.88	8
Balic ^a^ [38]	2009	Chile	ELISA	0.21±0.12	300	158/142	10.8±4.1	5.22	31.7	310	146/164	11.0±2.2	1.23	1.08	8
Machnica[39]	2014	Poland	ELISA	5.09±1.97	52	19/33	14.07±3.03	16.63	8.32	20	8/12	13.09±3.05	9.41	4.23	8
Alexandraki^b^ [7]	2008	Athens	ELISA	11.87±2.36	20	13/7	NR	0.67	0.27	34	17/17	NR	0.53	0.29	7
Romano ^a^ [40]	2001	Italy	ELISA	0.35±0.32	20	11/9	10.6±3.4	21.9	9.48	10	6/4	9.6±2.6	4.4	1.68	7
Araya[25]	2003	Chile	ELISA	12.0±5.9	15	8/7	22.2±3.1	5.7	1.5	14	5/9	25.1±3.7	1.3	0.2	8
Mitrovic ^c^ [41]	2011	Serbia	ELISA	20.01±8.78	76	32/44	35.24±11.09	0.65	0.37	30	12/18	38.10±12.97	0.2	0.11	8
Pham ^c^ [42]	2011	Germany	ELISA	0.1±0.40	90	62/28	43.2±4.45	2.4	0.5	41	16/25	47.7±3.38	1.8	0.35	7
Pertynska[22]	2010	Poland	ELISA	9.5±5.41	14	0/14	27.06±5.35	5.19	8.81	16	0/16	25.21±4.21	9.69	21.25	8
El-Samahy[24]	2015	Scoland	ELISA	6.1±1.6	32	12/20	12.4±2.6	17.6	1.9	30	11/19	13.4±1.9	6.5	1.22	8
Martos[43]	2006	Spain	ELISA	NR	20	10/10	7.34±0.88	3	3.1	40	20/20	8.16±0.46	1.4	1.2	7
Aguilera[44]	2015	Spain	ECMA	20.4±8.1	150	NR	38.6±8.1	7.5	2.4	50	NR	38.1±7.2	7.8	1.8	7
Gabbay[45]	2012	Brazil	FCM	NR	35	NR	13.0±5.0	13.8	26.9	25	NR	13.6±5.4	0.11	0.38	6
Lo[23]	2004	Taiwan	ELISA	NR	58	22/36	10.98±4.61	11.15	14.34	33	16/17	10.06±4.90	14.22	17.92	8
Abdel[46]	2001	Egypt	ECMA	5.48±3.22	15	8/7	13.71±4.26	14	10.67	15	8/7	13.21±3.20	6.61	1.97	8
Lechleitner[21]	2000	Austria	ELISA	12.8± 8.1	29	29/0	31.7±6.0	19.9	8.4	24	NR	30.5± 6.8	11.1	5.8	7
Lechleitner[21]	2000	Austria	ELISA	8.1±7.7	15	0/15	29.3±6.4	18.3	5.2	24	NR	30.5± 6.8	11.1	5.8	7
Lv[47]	2013	China	ELISA	0.16±0.08	33	18/15	12±4	27	14	27	15/12	11±4	26	6	8

FCM, flow cytometry method; ELISA, enzyme-linked immunosorbent assay; ECMA, enzyme chemiluminescence immunometric assay; SZ, sample size; M/F, male/female; SD, standard deviation; NR, not report.

^a^Data converted from median (range).

^b^SD data converted from SE.

^c^Data converted from median (interquartile range).

### Results of meta-analysis and subgroup analysis

**Fig 2:**
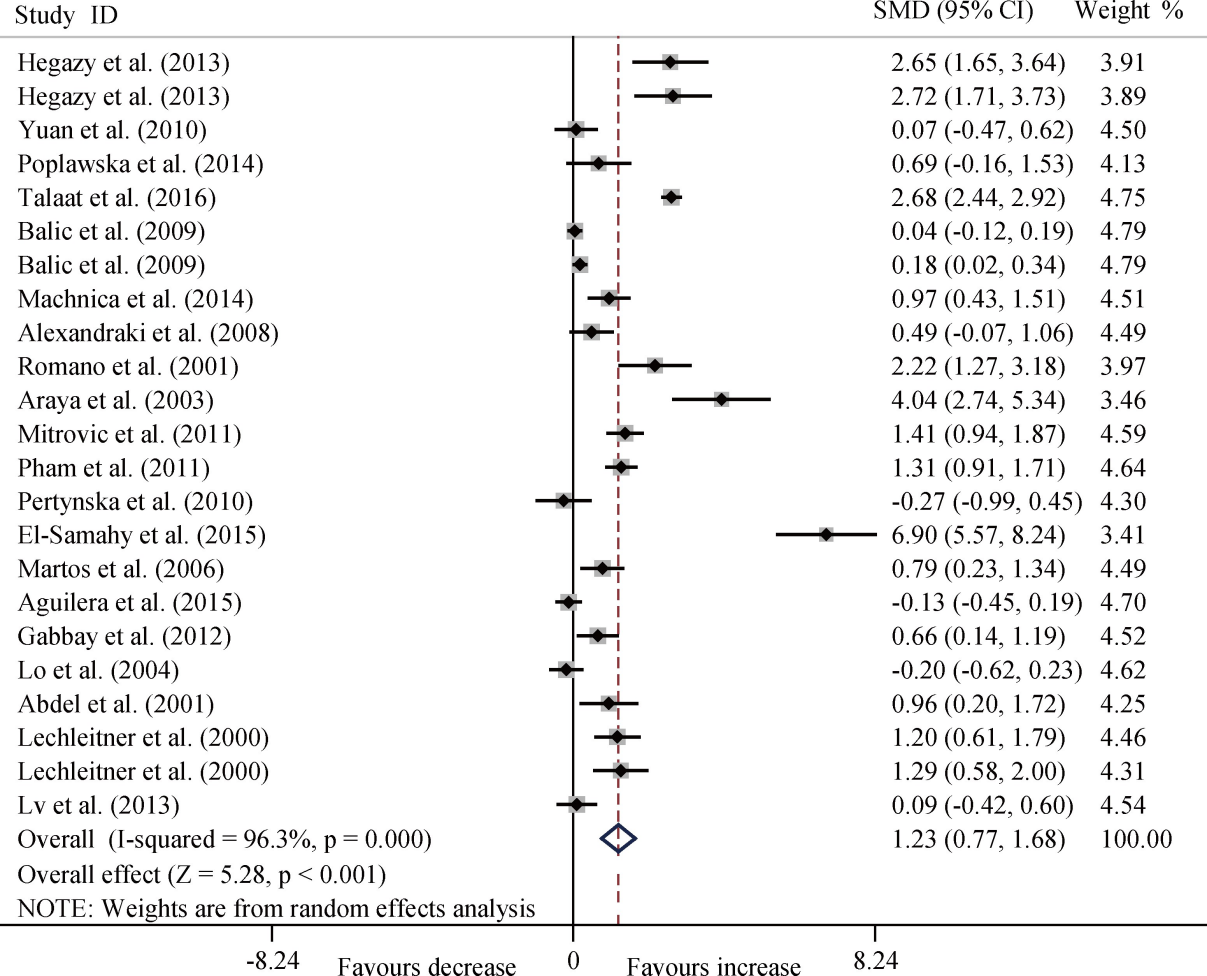
Forest plots for the level of serum TNF-α between T1DM patients and healthy controls with random effects model (SMD, 1.23, 95% CI, 0.77 to 1.68, *P* < 0.001).

The T1DM patients had significantly increased serum TNF-α level compared with the controls (SMD, 1.23; 95% CI, 0.77 to 1.68; *P* < 0.001) (Fig 2) and significant heterogeneity (*P* < 0.001) existed in this meta-analysis.

**Fig 3:**
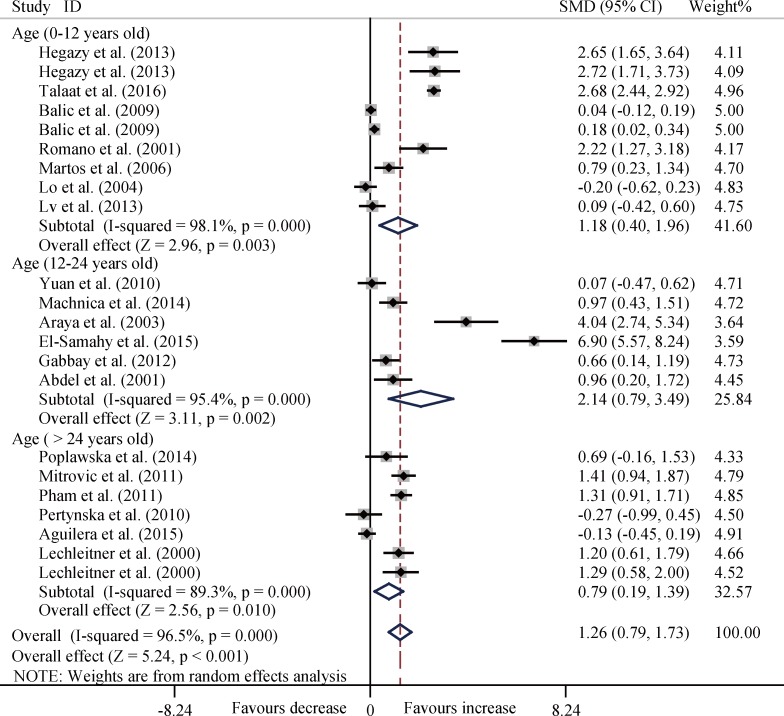
Forest plots about subgroup analysis for the level of TNF-α between T1DM patients and controls with random effects model among different age groups. Age, 0–12 years old, SMD, 1.18, 95% CI, 0.40 to 1.96, *P* = 0.003; Age, 12–24 years old, SMD, 2.14, 95% CI, 0.79 to 3.49, *P* = 0.002; Age, > 24 years old: SMD, 0.79, 95% CI, 0.19 to 1.39, *P* = 0.010; Overall, SMD, 1.26, 95% CI, 0.79 to 1.73, *P* < 0.001.

**Fig 4:**
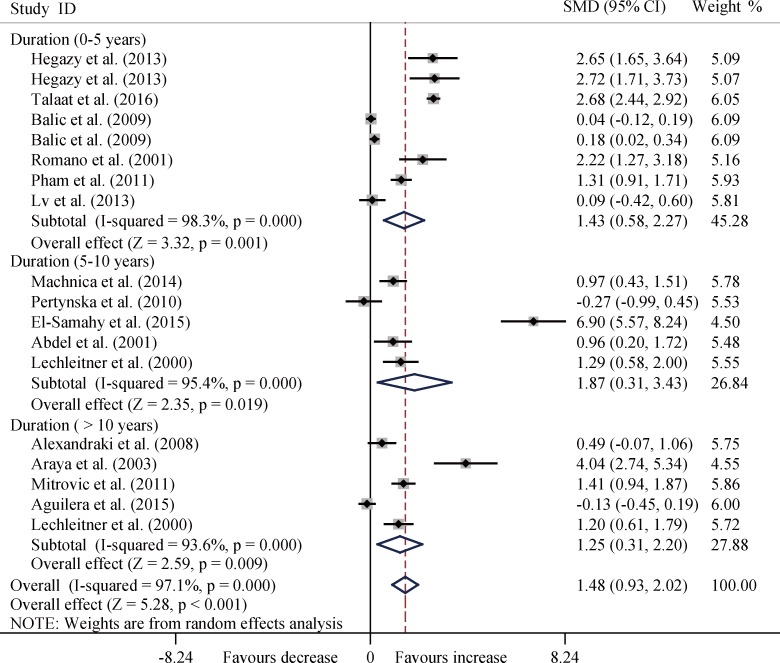
Forest plots about subgroup analysis for the level of TNF-α between T1DM patients and controls with random effects model among different disease duration groups. Duration, 0–5 years, SMD, 1.43, 95% CI, 0.58 to 2.27, *P* = 0.001; Duration, 5–10 years, SMD, 1.87, 95% CI, 0.31 to 3.43, *P* = 0.019; Duration, > 10 years, SMD, 1.25, 95% CI, 0.31 to 2.20, *P* = 0.009; Overall, SMD, 1.48, 95% CI, 0.93 to 2.02, *P* < 0.001.

**Fig 5:**
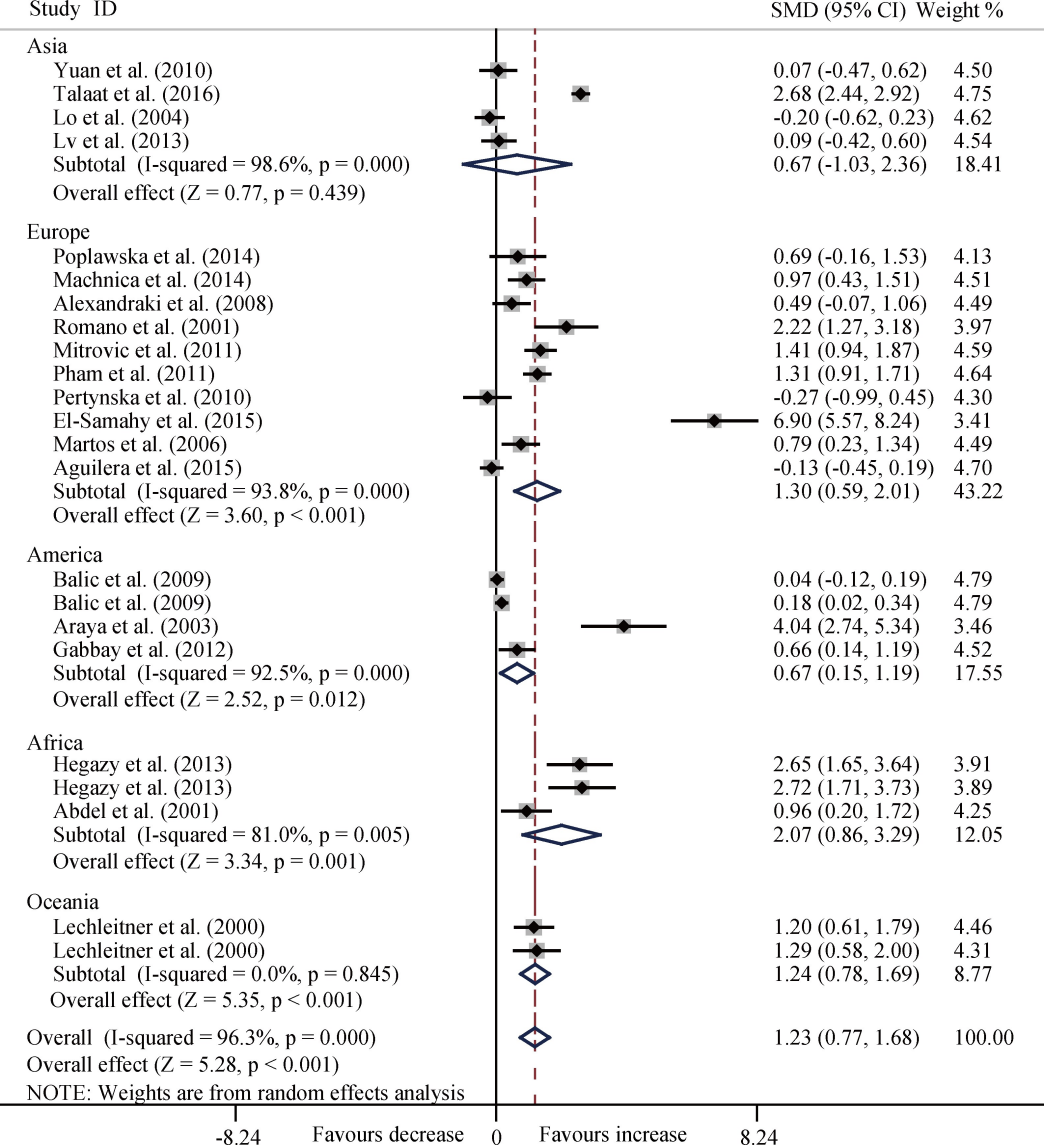
Forest plots about subgroup analysis for the level of TNF-α between T1DM patients and controls with random effects model among different ethnicity groups. Asia, SMD, 0.67, 95% CI, -1.03 to 2.36, *P* = 0.439; Europe, SMD, 1.30, 95% CI, 0.59 to 2.01, *P* < 0.001; America, SMD, 0.67, 95% CI, 0.15 to 1.19, *P* = 0.012; Africa, SMD, 2.07, 95% CI, 0.86 to 3.29, *P* = 0.001; Oceania, SMD, 1.24, 95% CI, 0.78 to 1.69, *P* < 0.001; Overall, SMD, 1.23, 95% CI, 0.77 to 1.68, *P* < 0.001.

In the subgroup analysis by age, disease duration, and ethnicity, T1DM patients consistently had significantly increased levels of TNF-α among the three age groups (all *P* < 0.01) (Fig 3), and three disease duration groups (all *P* < 0.05) (Fig 4), and four ethnicity subgroups (all *P* < 0.05) except Asia group (SMD, 0.67; 95%CI, -1.03 to 2.36; *P* = 0.439) (Fig 5).

### Regression analysis

Regression analysis was used to explore the source of high heterogeneity of age, disease duration, and ethnicity for the serum level of TNF-α, and the results displayed as follows: age (t = -0.42; 95% CI, -1.035 to 0.689; *P* = 0.680), disease duration (t = -0.06; 95% CI, -1.091 to 1.036; *P* = 0.957), and ethnicity of patients (t = 0.64; 95% CI, -0.298 to 0.567; *P* = 0.526), which indicated that age, disease duration, and ethnicity of patients were not significant impact factors for the high heterogeneity in this meta-analysis.

### Sensitivity analysis

**Fig 6:**
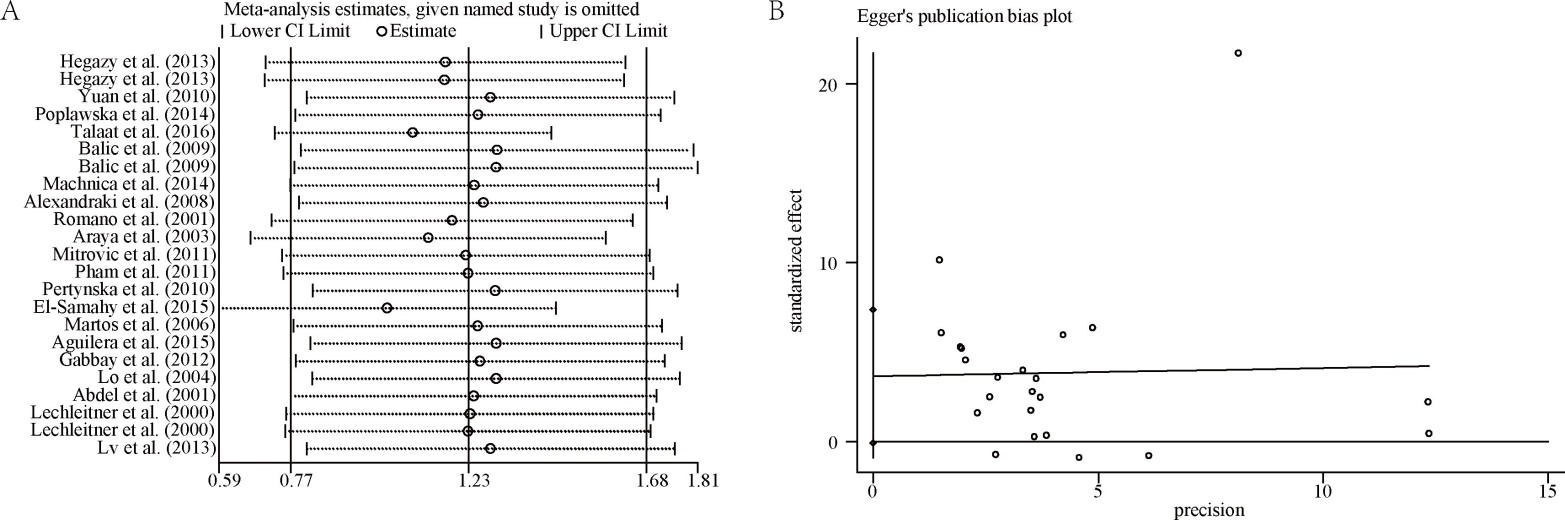
Sensitivity analysis and publication bias about the serum TNF-α level in T1DM. A: sensitivity analysis; B: publication bias, *t* = 2.04; *P* = 0.054; 95% CI, -0.074 to 7.370.

We first performed sensitivity analysis by excluding individual studies and found the results remaining consistent (Fig 6A). Next, sensitivity analysis was conducted by selecting studies with high NOS score (≥) or excluding studies with high risk of bias and we found that all the outcomes still had no significant changes. Otherwise, fixed effects model was chosen to pool the data, all the results were similar to those generated by random effects model.

### Publication bias

Egger’s test showed that no publication bias existed in this meta-analysis (t = 2.04; *P* = 0.054; 95% CI, -0.074 to 7.370) (Fig 6B).

## Discussion

TNF-α is highly involved with macrophage activation and increased serum TNF-α level have been observed in insulin resistance stages and diabetes mellitus development [48], however, inconsistency still existed and no meta-analysis was conducted about the change of serum TNF-α level in T1DM patients. The results of this study clearly demonstrate that T1DM patients had significantly elevated serum level of TNF-α and significant correlation existed between TNF-α level and patients’ age, disease duration and ethnicity.

TNF-α level may play an important role and many factors may contribute to the serum TNF-α level in diabetes. TNF-α, as the major physiological and pathophysiological regulators of vascular adhesion molecules, is a key proinflammatory cytokine with widespread metabolic effects, and directly regulate the production of several cardiovascular risk factors [49, 50]. TNF-α via effects on soluble intercellular adhesion molecule-1, may promote vascular adhesion, otherwise plasma levels of TNF-α are associated with dyslipidaemia and increase blood pressure, adding to vascular disease risk, besides, the actions of TNF-α is probably modified by altered production of soluble receptors in type 1 diabetic patients [51]. Increased TNF-α and interleukine-6 (IL-6) levels through metabolic control exist in types 1 and 2 diabetic patients, which suggest that the control of diabetes improves the capacity of activation and maintenance of these pro-inflammatory cytokines [52–54]. Other study [21] demonstrated TNF-α levels were elevated in T1DM which was correlated positively with HbA1c and inversely with HDL cholesterol levels. In addition, a significant relationship between TNF-α levels and both BMI and WHR also was observed in analysis of the combined groups [51].

Owning the various biological effects, TNF-α has been proved to have certain catabolic effects on fat cells, and neutralization of TNF-α in obese rats causes a significant increase in the peripheral uptake of glucose in response to insulin, which indicates an important role in the insulin resistance and diabetes that often accompany obesity [55]. TNF-α, through increasing the activities of the NF-κB transcriptional factor [56, 57], protein kinase C [58], amino terminal kinase and inhibitor kinase, could cause serine/threonine phosphorylation of the insulin receptor substrate, interfere with normal phosphorylation of tyrosine, and weaken signal transduction of insulin, resulting in insulin resistance [36], otherwise, TNF-α may be result in the destruction of pancreatic beta cells and lead to the development of T1DM [59].

Age, disease duration and ethnicity of T1DM patients were focused on in this study. In a previous study [60], serum TNF-α level was not associated with the presence and severity of microalbuminuria, otherwise, the level of urinary TNF-α was only significantly influenced by albumin-creatinine ratio (ACR), although other factors had been included in the multivariate analysis: age, duration of diabetes, BMI, history of cardiovascular disease, presence of retinopathy, hypertension, and HbA1c levels. Lo, H. C., et al. [23] found that serum concentrations of TNF-α had no significant change in type 1 diabetic children compared with healthy siblings between different age groups (1–6 years old group, 6–12 years old group and 12–18 years old group), which was inconsistent with our findings. Small sample size and different statistical approach maybe illuminate the phenomenon. Ethnicity maybe another impact factor for the level of TNF-α and no significant change existed in Asia populations. In this multifactorial disease, Asia populations with varied geographic distribution, linked to climate, diet, lifestyle and economic status may contribute to the discrepancy.

Significant heterogeneity still existed after subgroups analysis and regression analysis indicated that these factors were not potential sources for the high heterogeneity, and we assume that sources of heterogeneity may be attributed to the diversity in design, sample sizes, measurement errors and so on. Otherwise, sensitivity analysis indicated the results were stable and no publication bias existed in this meta-analysis.

Several limitations should be considered when cautiously interpreting the results. Firstly, we could not conduct further subgroup analyses such as by gender, body weight and other factors because of insufficient original data. Secondly, only reports in English and reports in Chinese were chosen and eligible studies might have not been unpublished or published in other languages. Small sample size, limited statistical power and high heterogeneity of the included studies could also influence the results. Furthermore, some reports included in this meta-analysis failed to disclose the status of diabetic complications, which may have impact on the results. All these limitations should be kept in mind when interpreting the findings.

In summary, the present meta-analysis indicates that compared with the healthy controls, the T1DM patients have significantly increased serum level of TNF-α. The role of TNF-α in the development of diabetes and diabetic complications warrant future investigation.

## Supporting information

S1 Appendix The references of the data source with DOI and access no. (DOC)

S1 Table PRISMA 2009 checklist. (DOC)
