# Supplementary material for: The change of serum tumor necrosis factor alpha in patients with type 1 diabetes mellitus: A systematic review and meta-analysis
Source: PLoS One. 2017 Apr 20;12(4):e0176157. doi: 10.1371/journal.pone.0176157 (PMC5398633; doi:10.1371/journal.pone.0176157)
Supplement: S1 Appendix — (DOC) [file pone.0176157.s001.doc]

Appendix: The references of the data source with DOI and access no

1. Poplawska-Kita A, Siewko K, Szpak P, Krol B, Telejko B, Klimiuk PA, et al. Association between type 1 diabetes and periodontal health. Advances in medical sciences. 2014;59(1):126-31. Epub 2014/05/07. doi: 10.1016/j.advms.2014.01.002. PubMed PMID: 24797988.

7. Alexandraki KI, Piperi C, Ziakas PD, Apostolopoulos NV, Makrilakis K, Syriou V, et al. Cytokine secretion in long-standing diabetes mellitus type 1 and 2: associations with low-grade systemic inflammation. Journal of Clinical Immunology. 2008;28(4):314-21. Epub 2008/01/29. doi: 10.1007/s10875-007-9164-1. PubMed PMID: 18224429.

21. Lechleitner M, Koch T, Herold M, Dzien A, Hoppichler F. Tumour necrosis factor-alpha plasma level in patients with type 1 diabetes mellitus and its association with glycaemic control and cardiovascular risk factors. Journal of Internal Medicine. 2000;248(1):67-76. Epub 2000/08/18. doi: 10.1046/j.1365-2796.2000.00705.x. PubMed PMID: 10947883.

22. Pertynska-Marczewska M, Glowacka E, Grodzicka A, Sobczak M, Cypryk K, Wilczynski JR, et al. Profile of peripheral blood neutrophil cytokines in diabetes type 1 pregnant women and its correlation with selected parameters in the newborns. American Journal of Reproductive Immunology (New York, NY : 1989). 2010;63(2):150-60. Epub 2009/12/31. doi: 10.1111/j.1600-0897.2009.00775.x. PubMed PMID: 20039861.

23. Lo HC, Lin SC, Wang YM. The relationship among serum cytokines, chemokine, nitric oxide, and leptin in children with type 1 diabetes mellitus. Clinical Biochemistry. 2004;37(8):666-72. Epub 2004/08/11. doi: 10.1016/j.clinbiochem.2004.02.002. PubMed PMID: 15302608.

24. El-Samahy MH, Adly AA, Ismail EA, Salah NY. Regulatory T cells with CD62L or TNFR2 expression in young type 1 diabetic patients: relation to inflammation, glycemic control and micro-vascular complications. Journal of Diabetes and Its Complications. 2015;29(1):120-6. Epub 2014/08/13. doi: 10.1016/j.jdiacomp.2014.07.004. PubMed PMID: 25113439.

25. Araya AV, Pavez V, Perez C, Gonzalez F, Columbo A, Aguirre A, et al. Ex vivo lipopolysaccharide (LPS)-induced TNF-alpha, IL-1beta, IL-6 and PGE2 secretion in whole blood from Type 1 diabetes mellitus patients with or without aggressive periodontitis. European Cytokine Network. 2003;14(3):128-33. Epub 2003/12/06. PubMed PMID: 14656685.

35. Hegazy SK, Tolba OA, Mostafa TM, Eid MA, El-Afify DR. Alpha-lipoic acid improves subclinical left ventricular dysfunction in asymptomatic patients with type 1 diabetes. The Review of Diabetic Studies : RDS. 2013;10(1):58-67. Epub 2013/11/01. doi: 10.1900/rds.2013.10.58. PubMed PMID: 24172699; PubMed Central PMCID: PMCPMC3932072.

36. Yuan T, Zhao WG, Sun Q, Fu Y, Dong YY, Dong YX, et al. Association between four adipokines and insulin sensitivity in patients with obesity, type 1 or type 2 diabetes mellitus, and in the general Chinese population. Chinese Medical Journal. 2010;123(15):2018-22. Epub 2010/09/08. doi: 10.3760/cma.j.issn.0366-6999.2010.15.012. PubMed PMID: 20819535.

37. Talaat IM, Nasr A, Alsulaimani AA, Alghamdi H, Alswat KA, Almalki DM, et al. Association between type 1, type 2 cytokines, diabetic autoantibodies and 25-hydroxyvitamin D in children with type 1 diabetes. Journal of Endocrinological Investigation. 2016. Epub 2016/08/20. doi: 10.1007/s40618-016-0514-9. PubMed PMID: 27541155.

38. Balic I, Angel B, Codner E, Carrasco E, Perez-Bravo F. Association of CTLA-4 polymorphisms and clinical-immunologic characteristics at onset of type 1 diabetes mellitus in children. Human Immunology. 2009;70(2):116-20. Epub 2009/01/13. doi: 10.1016/j.humimm.2008.12.007. PubMed PMID: 19136037.

39. Machnica L, Deja G, Polanska J, Czupryniak L, Szymanska-Garbacz E, Loba J, et al. Blood pressure disturbances and endothelial dysfunction markers in children and adolescents with type 1 diabetes. Atherosclerosis. 2014;237(1):129-34. Epub 2014/09/23. doi: 10.1016/j.atherosclerosis.2014.09.006. PubMed PMID: 25238220.

40. Romano M, Pomilio M, Vigneri S, Falco A, Chiesa PL, Chiarelli F, et al. Endothelial perturbation in children and adolescents with type 1 diabetes: association with markers of the inflammatory reaction. Diabetes Care. 2001;24(9):1674-8. Epub 2001/08/28. doi:org/10.2337/diacare.24.9.1674. PubMed PMID: 11522718.

41. Mitrovic M, Ilic T, Stokic E, Paro JN, Naglic DT, Bajkin I, et al. Influence of glucoregulation quality on C-reactive protein, interleukin-6 and tumor necrosis factor-alpha level in patients with diabetes type 1. Vojnosanitetski Pregled. 2011;68(9):756-61. Epub 2011/11/04. doi: 10.2298/VSP1109756M. PubMed PMID: 22046880.

42. Pham MN, Hawa MI, Pfleger C, Roden M, Schernthaner G, Pozzilli P, et al. Pro- and anti-inflammatory cytokines in latent autoimmune diabetes in adults, type 1 and type 2 diabetes patients: Action LADA 4. Diabetologia. 2011;54(7):1630-8. Epub 2011/02/25. doi: 10.1007/s00125-011-2088-6. PubMed PMID: 21347621.

43. Martos-Moreno GA, Barrios V, Soriano-Guillen L, Argente J. Relationship between adiponectin levels, acylated ghrelin levels, and short-term body mass index changes in children with diabetes mellitus type 1 at diagnosis and after insulin therapy. European Journal of Endocrinology / European Federation of Endocrine Societies. 2006;155(5):757-61. Epub 2006/10/26. doi: 10.1530/eje.1.02273. PubMed PMID: 17062893.

44. Aguilera E, Serra-Planas E, Granada ML, Pellitero S, Reverter JL, Alonso N, et al. Relationship of YKL-40 and adiponectin and subclinical atherosclerosis in asymptomatic patients with type 1 diabetes mellitus from a European Mediterranean population. Cardiovascular Diabetology. 2015;14:121. Epub 2015/09/19. doi: 10.1186/s12933-015-0287-z. PubMed PMID: 26382922; PubMed Central PMCID: PMCPMC4574547.

45. Gabbay MA, Sato MN, Duarte AJ, Dib SA. Serum titres of anti-glutamic acid decarboxylase-65 and anti-IA-2 autoantibodies are associated with different immunoregulatory milieu in newly diagnosed type 1 diabetes patients. Clinical and Experimental Immunology. 2012;168(1):60-7. Epub 2012/03/06. doi: 10.1111/j.1365-2249.2011.04538.x. PubMed PMID: 22385239; PubMed Central PMCID: PMCPMC3390495.

46. Abdel Aziz MT, Fouad HH, Mohsen GA, Mansour M, Abdel Ghaffar S. TNF-alpha and homocysteine levels in type 1 diabetes mellitus. Eastern Mediterranean Health Journal = La revue de sante de la Mediterranee orientale = al-Majallah al-sihhiyah li-sharq al-mutawassit. 2001;7(4-5):679-88. Epub 2004/08/31. PubMed PMID: 15332766.

47. Lv L, Liu JY, Ma J, Lin SX, Huang L. [Factors associated with Th1 cytokine disorders in children with newly diagnosed type 1 diabetes]. Zhongguo Dang Dai Er Ke Zazhi = Chinese Journal of Contemporary Pediatrics. 2013;15(1):50-2. Epub 2013/01/23. doi: 10.7499/j.issn.1008－8830.2013.01.013. PubMed PMID: 23336169.
